# A case report of intraventricular and intrathecal tigecycline infusions for an extensively drug-resistant intracranial *Acinetobacter baumannii* infection

**DOI:** 10.1097/MD.0000000000015139

**Published:** 2019-04-12

**Authors:** Zi-Wei Deng, Jin Wang, Cheng-Feng Qiu, Yi Yang, Zhi-Hua Shi, Jian-Liang Zhou

**Affiliations:** a Department of Clinical Pharmacy, The First People's Hospital of Huaihua; b Huaihua Center for Evidence-based Medicine and Clinical Research; c Department of Infectious Disease; d Department of Radiology, The First People's Hospital of Huaihua, Huaihua, Hunan, China.

**Keywords:** clinical pharmacist, intracranial infection, intraventricular, tigecycline

## Abstract

**Rationale:**

The treatment of intracranial *Acinetobacter baumannii* infections is made difficult by multidrug-resistance poor drug penetration through the blood-brain barrier (BBB). Although tigecycline appears to be effective against *A baumannii*, it is only administered intravenously because it does not readily cross the BBB. The addition of intraventricular (IVT) or intrathecal infusions of tigecycline could revolutionize clinical therapy for intracranial *A baumannii* infections. However, there are few reports on the successful use of such treatments.

**Patient concerns:**

We report the case of a 17-year-old male who presented with high fever and neck rigidity after intracranial drainage.

**Diagnosis:**

Intracranial infection with extensively drug-resistant *A baumannii* after intracranial drainage.

**Interventions:**

On the advice of a clinical pharmacist, the patient was administered intrathecal infusions of tigecycline after treatment failure with IVT tigecycline.

**Outcomes:**

The patient's body temperature returned to normal. Thereafter, the patient was in good clinical condition without signs of cerebrospinal fluid infection and tuberculosis.

**Lessons:**

However, when central nervous system infections fail IVT tigecycline, clinicians should consider changing to intrathecal tigecycline infusions rather than raising the dose of IVT tigecycline. In addition, the co-administration of tigecycline with other drugs that can penetrate the BBB should not be ruled out.

## 1 Introduction

*Acinetobacter baumannii* central nervous system (CNS) infections represent 3.6% to 11.2% cases of nosocomial intracranial infections caused by multidrug-resistant or extremely drug-resistant strains.^[1]^ The mortality rate of *A baumannii* CNS infections is as high as 71%,^[1]^ which suggests a dismal prognosis. Not many antibiotics are available for the treatment of intracranial extremely drug-resistant *A baumannii* (XDRAB) infections because most drugs cannot enter the CNS. Thus, although the incidence of neurotoxicity (up to 21.7%) limits its clinical use, intraventricular (IVT) or intrathecal infusion of colistin is the current treatment of choice.

Tigecycline is the first clinically available drug from the glycylcycline class of antibiotics. Several studies have proven its activity against *Acinetobacter* strains, including XDRAB.^[2]^ However, intravenous (IV) tigecycline is not recommended for the treatment of intracranial infections because of its limited ability to penetrate the blood-brain barrier (BBB; penetration rate ∼11%). A viable alternative for the treatment of intracranial infections would be IVT tigecycline, as it has a low incidence of CNS adverse reactions.^[3]^

In this case report, a clinical pharmacist fully participated in the pharmaceutical monitoring of 1 patient with an XDRAB intracranial infection. The pharmacist actively reviewed the literature to determine the dosage and feasibility of intracranial tigecycline infusions, as well as infusions of tigecycline to the lateral ventricle. Other options were also discussed with the clinician, and the resulting treatment ultimately controlled the patient's intracranial infection.

## 2 Consent

The patient signed informed consent for the publication of this case report and any accompanying images. Ethical approval of this study was waived by the ethics committee of The First People's Hospital of Huaihua because this is a case report and there was only 1 patient.

## 3 Case report

**Figure 1:**
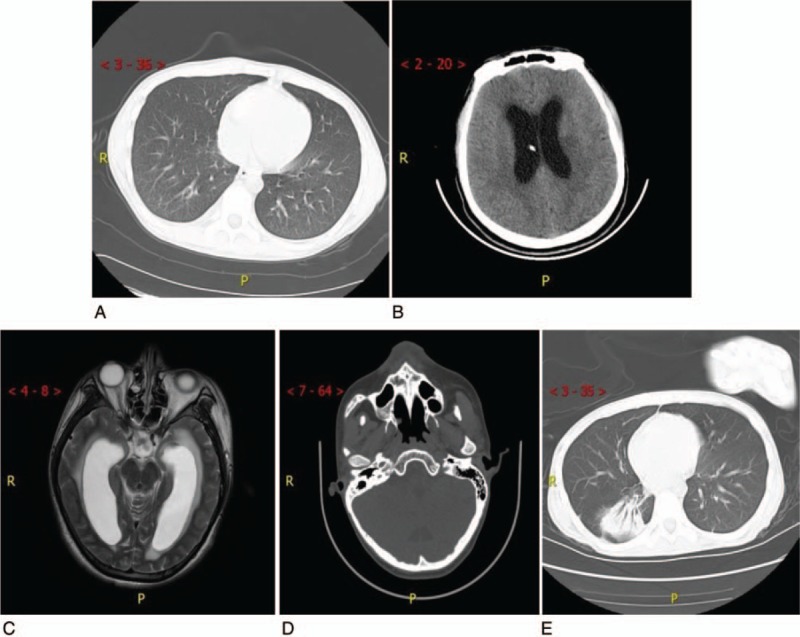
Computed tomography (CT) image after admission. (A) Nonenhanced pulmonary CT on Feb. 2, 2018. (B) Nonenhanced skull CT scan shows intracranial drainage with increased reduction of intracranial pressure. (C) Skull MRI upon first CSF culture shows XDRAB infection. (D) Nonenhanced skull CT scan after transfer to the rehabilitation unit. (E) Nonenhanced pulmonary CT scan after transfer to the rehabilitation unit. CSF = cerebrospinal fluid, CT = computed tomography, MRI = magnetic resonance imaging, XDRAB = extensively drug-resistant *Acinetobacter baumannii*.

A 17-year-old male was transferred to our hospital on Feb 2, 2018, for prolonged fever (1 month) and disturbances in consciousness lasting 3 days. Before illness he had been healthy, but upon admission, pulmonary computed tomography (CT) scans (Fig. 1[A]) and Xpert analysis of cerebrospinal fluid (CSF; positive for *Mycobacterium tuberculosis*) indicated tuberculous meningitis. After hospitalization, anti-tuberculosis drugs (rifampin, isoniazid, pyrazinamide, ethambutol, and linezolid) and low-dose dexamethasone were administered via intrathecal infusion; intracranial pressure was managed via dehydration, diuresis, and intracranial drainage (Fig. 1[B]).

Nine days after admission, the patient's mental status had not improved. Moist rales and sputum in the cavum oris had significantly increased, and the patient experienced a sudden onset of fever (40°C) with symptoms of restlessness and shortness of breath. His peripheral oxygen saturation dropped to 75%. Tracheal intubation was performed; IV piperacillin-tazobactam (4.5 g every 8 h) was administered for infection and the patient was transferred to the intensive care unit.

On the 24th day, a slight fever remained. Amikacin (0.5 g daily) was added to the patient's antibiotic regimen and he underwent a tracheotomy, but the low fever persisted (around 38°C). A moderate amount of yellow sticky phlegm was duly aspirated from an incision in his trachea.

On the 30th day, the high fever returned. The patient's temperature rose to 39.5°C and he began experiencing chills. His CSF turned yellow and obviously turbid. His white blood cell (WBC) count was13.8 × 10^9^/L, neutrophilic granulocyte percentage (NEUT%) was 87.10%. Results of routine CSF tests were as follows: cell count, 1560 × 10^6^/L; WBC, 310 × 10^6^/L; and acid-fast stain, negative. Empirical treatment with vancomycin (1 g every 12 h) and meropenem (1 g every8 h) combined with fosfomycin (4.0 g every 8 h) was initiated.

On the 36th day, the patient's CSF culture implied an XDRAB infection that was only sensitive to tigecycline (minimum inhibitory concentration [MIC] = 1 μg/mL). A magnetic image of the patient's skull indicated an intracranial infection (Fig. 1[C]). In light of the poor BBB permeability of tigecycline, off-label IVT injections were considered. A therapeutic schedule of IVT tigecycline was instituted with permission from members of the patient's family. A clinical pharmacist performed a literature search to determine the dosage. Per her recommendation, tigecycline was diluted in saline up to a total volume of 5 mL, and 4 mg of tigecycline was slowly injected into the patient's ventricular system every 12 hours. During that procedure, his drainage tube was temporarily closed for approximately 2 hours. Meanwhile, meropenem was changed to cefoperazone-sulbactam (3 g every 8 h), and IV tigecycline (47.5 mg twice a day) and fosfomycin (4 g every 8 h) were added to the patient's regimen.

On the 40th day, the patient's body temperature began to gradually decline; but on the 45th day, his temperature showed an upward trend with an apex of 38.5°C. His CSF culture suggested an XDRAB infection that was sensitive only to tigecycline (MIC = 2 μg/mL). The clinical pharmacist advised changing from IVT to intrathecal infusions of tigecycline (4 mg daily) rather than increasing the IVT tigecycline dosage and changing cefoperazone-sulbactam to IV meropenem (2 g every 8 h) using an optimized 2-step infusion process (1 g over 0.5 h, then 1 g over 2.5 h). The pharmacist also suggested that fosfomycin is infused in dual venous channels; this was adopted by the clinician.

On the 52nd day after admission, the patient's CSF culture suggested an XDRAB infection that was only sensitive to tigecycline (MIC = 1 μg/mL).

On the 55th day, the patient's temperature returned to normal. The results of routine CSF tests were as follows: cell count, 30 × 10^6^/L; WBC, 10 × 10^6^/L.

On the 75th day, the patient's cultures were negative for 3 consecutive tests, and CSF acid-fast stains were negative. At that point, the antibiotics were discontinued, but the patient remained on anti-tuberculosis therapy. He was transferred to the rehabilitation unit for functional restoration.

At the 4-month follow-up, the patient was in good clinical condition without signs of CSF infection and tuberculosis (Fig. 1[D, E]).

**Figure 2:**
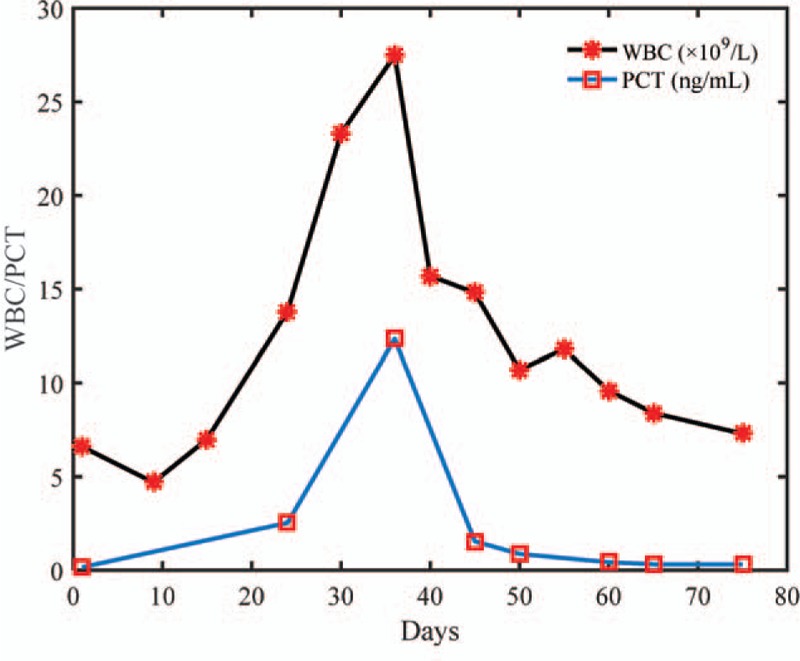
Changes in serum leukocyteand procalcitonin levelsover the course of treatment. PCT = procalcitonin.

**Figure 3:**
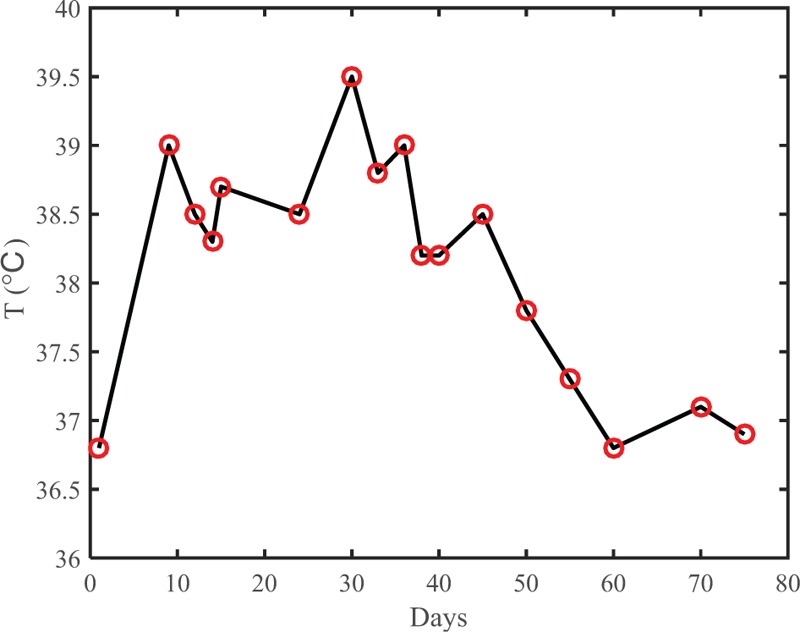
Changesin body temperature over the course of treatment.

Changes over the course of the patient's treatment are shown in the following table and figures: Table 1 shows changes in the CSF; Figure 2 shows changes in serum leukocyte and procalcitonin levels; Figure 3 shows changes in the patient's body temperature.

**Table 1:**
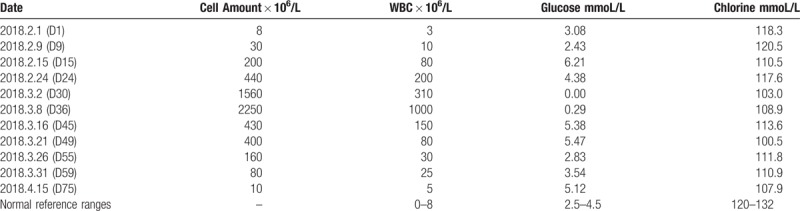
Laboratory results for cerebral spinal fluid over the course of treatment.

## 4 Discussion

### 4.1 Analysis of IVT and intrathecal infusion of tigecycline for the treatment of intracranial XDRAB infection

Because IVT antimicrobials bypass the blood-CSF barrier, they can attain high/moderate CSF concentrations that are greater than those attained by the use of IV antimicrobials.^[4]^ Thus, published guidelines recommend that IVT antimicrobials be considered in cases that are unresponsive to IV antimicrobials alone, or when the CSF concentrations of IV antimicrobials do not meet their respective MICs, especially with regard to multidrug-resistant organisms.^[5]^

The first successful use of IVT tigecycline for the treatment of XDRAB meningitis was reported in 2016.^[1]^ In that case, IVT tigecycline was initiated at a dose of 2 mg daily while continuing IV tigecycline; meropenem and vancomycin were discontinued. Unfortunately, fever recurred after 10 days of treatment. The IVT tigecycline dose was raised to 2 mg every 12 h. After 1 month of treatment, the ventricular catheter was removed and the antibiotic therapy was discontinued.

In another case, Fang et al^[6]^ initiated IVT tigecycline for XDRAB meningitis at a dose of 3 mg daily. Meanwhile, IV tigecycline (97 mg every 12 h) and cefoperazone-sulbactam (3 g every 12 h) were administered. After 6 days of treatment, the CSF culture remained positive. Then, IVT tigecycline was added to a maximum dose of 4 mg every 12 hours. After a half month of treatment, the patient's symptoms were alleviated.

In 2017, a case of carbapenem-resistant *Klebsiella pneumonia* after craniotomy was reported in which 3 different doses of IVT tigecycline were administered as follows: 1 mg, 5 mg, and 10 mg every 12 hours in combination with IV tigecycline infusion.^[7]^ There were no obvious drug adverse reactions. In another case^[1]^, intrathecal tigecycline infusion was used to treat *Neisseria meningitis*, but no details on efficacy were presented in it.

The XDRAB and carbapenem-resistant *K pneumonia* infections in all the above reports were treated via craniotomy or ventricular catheters. Those cases demonstrate that antimicrobials might be necessary when patients do not respond to IV antimicrobials alone. In our patient's case, we adopted a tigecycline IVT dosage of 4 mg every 12 hours based on an MIC of 1 μg/mL. When that treatment failed, the dosage of tigecycline was not increased because the safety such an increase was unclear. Instead, we changed the route of tigecycline administration from IVT infusions to intrathecal infusions of 4 mg daily. Thereafter, the patient was in good clinical condition without signs of CSF infection. In our study, the change to intrathecal tigecycline infusions resulted in the best possible outcome.

### 4.2 Antibiotic selection for intracranial XDRAB infection in combination with tigecycline

Blood-CSF and BBB permeabilities to sulbactam increase from 1% to 33%,^[8]^ because the antibacterial activity of sulbactam is directly related to its concentration. The recommended dose of sulbactam for the treatment of severe *A baumannii* infection is 9 to 12 g daily.^[2]^ However, the dosage of sulbactam was just 4.5 g daily in our patient's case. The clinical pharmacist analyzed that dosage and determined that sulbactam was ineffective because the dosage was too low.

One issue that was considered is BBB penetration. Fortunately, the BBB is highly permeable to carbapenems. The MIC values of carbapenems against moderately susceptible bacteria are similar to carbapenem CSF concentrations attained with uninflamed meningitis.^[9]^ In addition, carbapenems have few CNS adverse drug reactions. Thus, they are useful for the treatment of intracranial *A baumannii* infections. In this case, the clinical pharmacist suggested discontinuing cefoperazone-sulbactam and replacing it with meropenem at a dosage of 2.0 g IV every 8 hours in a 2-step method to extend infusion time.

The second issue was drug resistance. The mechanism of drug resistance ingram-negative bacilli is complex and likely due to the production of β-lactamase, biofilm, active efflux, and so on. One of the mechanisms by which pan drug-resistant *Pseudomonas aeruginosa* and pan drug-resistant *A baumannii* strains maintain resistance is by producing biofilm that prevents or inhibits antibiotics from entering cells. Sombat et al reported that fosfomycin significantly reduced the MIC values of colistin against 12 strains of pan drug-resistant *A baumannii* and pan drug-resistant *P aeruginosa* isolated from patients admitted to a 550-bed private hospital in Bangkok and Thailand.^[10]^ Moreover, fosfomycin CSF concentrations are higher than the MICs of fosfomycin against susceptible pathogens in both inflamed and uninflamed meningitis. Thus, combining fosfomycin with relevant antimicrobial agents for the treatment of drug-resistant *A baumannii*is becoming more common. In this case, the pharmacist suggested continuing fosfomycin and dual-channel venous infusions of meropenem in combination with IVT and IV tigecycline.

## 5 Conclusions

We found that IVT was a convenient route of drug administration, especially during intracranial drainage. Although a few case reports on tigecycline IVT administration have noted a scant number of adverse drug reactions, the true frequency of adverse effects may emerge only after long-term exposure in a large number of patients. In addition, information on the safety of intrathecal tigecycline infusion is lacking and requires further evaluation. Hence, IVT tigecycline should only be considered as an alternative strategy. In cases of IVT failure, an increase in dosage is not recommended; rather, the treatment should be changed to intrathecal tigecycline infusion. In addition, clinicians should consider combining tigecycline with IV drugs that can penetrate the BBB with relative ease.

## Acknowledgments

The authors would like to thank Dr Li-an Bian (Changsha University of Science & Technology, Hunan, China) for repeatedly revising this paper.

## Author contributions

**Data curation:** Zi-Wei Deng, Zhi-Hua Shi.

**Methodology:** Zi-Wei Deng.

**Project administration:** Jin Wang, Jian-Liang Zhou.

**Software:** Zi-Wei Deng.

**Supervision:** Jian-Liang Zhou.

**Writing – original draft:** Zi-Wei Deng.

**Writing – review & editing:** Zi-Wei Deng, Cheng-Feng Qiu, Jian-Liang Zhou, Yi Yang.
